# Examining ultra-processed food consumption and attention deficit/hyperactivity disorder symptoms in children living in Kuwait

**DOI:** 10.3389/fnut.2026.1830606

**Published:** 2026-07-14

**Authors:** Saleh Alali, Wed Al-Otaibi, Sara Alotaibi, Haya Altuwaijri, Shaikha Alaradh, Shoug Althfeer

**Affiliations:** Nutrition and Food Science Department, Kuwait University, Kuwait City, Kuwait

**Keywords:** ADHD, children behavioral development, food additives, SNAP-IV parent rating scale, ultra-processed foods

## Abstract

**Introduction:**

The meteoric rise of ultra-processed foods (UPFs) in our diets has become a global public health concern. In Kuwait, globalization has led to a shift in dietary patterns increasing reliance on UPFs, particularly among children. UPFs have been shown to stimulate the brain’s reward-system making them especially relevant to attention-deficit/hyperactivity disorder (ADHD) related behaviors such as impulsivity and reward-seeking. Although determining the direction of the relationship between ADHD and UPF consumption is difficult, limited evidence, particularly in Kuwait, has examined this association during childhood.

**Methods:**

A cross-sectional study using the SNAP-IV and NOVA based ultra-processed food frequency questionnaire were used to examine the relationship between ultra-processed food intake and ADHD behavioral outcomes in Kuwaiti children between the ages 6 and 12.

**Results:**

A total of *N* = 159 were included (mean age 8.58 years and 59.1% male) in the final sample. After adjusting for age, a significant positive association was found (*ρ* = 0.576, *p* < 0.001) between total ADHD symptom severity and UPF consumption, hyperactivity showed the strongest association in the total sample followed by inattention. Boys (*n* = 94, *ρ* = 0.665, *p* < 0.001) had stronger associations compared to girls (*n* = 65, *ρ* = 0.439, *p* < 0.001) with hyperactivity having the strongest link among boys and inattention having the strongest correlation among girls.

**Discussion:**

These findings highlight the harmful behavioral association between ultra-processed food consumption and child development. Higher intake of synthetic ingredients is associated with increased ADHD symptom severity in this general population of Kuwaiti children. These results along with growing evidence highlight the need for urgent interventions. Public policy action is needed to restrict and regulate ultra-processed foods and artificial ingredients in Kuwait.

## 1 Introduction

Attention-Deficit/Hyperactivity Disorder (ADHD) is a neurodevelopmental disorder typically diagnosed during childhood and adolescence. The hallmarks of the disorder are heightened presentations of inattention and/or hyperactivity/impulsivity that interfere with functional development (1). Disruptive behavioral patterns associated with ADHD hinder numerous facets of social, academic, and cognitive functioning, leading to significantly reduced health-related quality of life compared to their peers (2).

The global prevalence of ADHD is estimated to be 7.2%, with recent data from the Arab Gulf region estimating the prevalence at 5.9% (3, 4). Although the etiology of ADHD is not fully elucidated, current evidence points to a combination of genetic and environmental factors that may be involved. The role of nutrition has also been investigated as a key modifiable factor in both the etiology and management of the disorder. Dietary interventions such as the Mediterranean diet and the elimination diet have demonstrated moderate success in reducing symptom severity (5–7). In addition, deficiencies in vitamin D and minerals such as iron and zinc have also been linked to poorer behavioral outcomes (8). However, evidence linking nutrition to either the etiology or pathology of ADHD remains inconclusive (9–11).

For example, studies investigating sugar intake have found no association between consumption during childhood and the incidence of ADHD, while other studies have found associations during both childhood and the prenatal period (11–14). However, current literature suggests a more complex relationship between ADHD and diet, affecting multiple pathways and mechanisms such as the gut-brain axis and neuroinflammation that may alter neurological development (15, 16). Beyond sugar intake, ultra-processed foods (UPFs) have gained attention for their implications in gut dysbiosis and cognitive functioning, especially during child development (15, 17). The NOVA-based classification system defines UPFs as extensively industrially processed foods and beverages, often made from refined and artificial ingredients with limited or void of nutritional value (18). Manufacturers design UPFs to be highly palatable, profitable, and convenient (19). Due to their accessibility, UPFs have become an unavoidable part of the modern diet, potentially displacing nutrient-dense staples and whole foods. UPF consumption has rapidly become a global health concern due to its documented negative effects on human health, especially during early child development (20–22). Exposure to UPFs may start as early as infancy, partly due to convenience, but also due to child-centered marketing of these products (23, 24). UPFs have been shown to stimulate the brain’s reward-system making them especially relevant to ADHD-related behaviors such as impulsivity and reward-seeking (25–27). Petroleum-derived food additives are commonly found in UPFs to enhance color, texture, and shelf-life across a wide-range of products such as, candies, baked goods, and brightly colored soft-drinks (18). In the United States, the Food and Drug Administration allows petroleum-based artificial food colorings to contain allowable levels of heavy metals such as lead (Pb), arsenic (As), and mercury (Hg) as residual contaminants from manufacturing (28–30). While only trace amounts are permitted, a growing body of evidence suggests that chronic low levels of exposure to heavy metals, particularly lead (Pb), is associated with impaired neurodevelopment and has been specifically linked to ADHD symptoms in children (28, 31). Although defining the directionality of the relationship between ADHD and UPF consumption is difficult, limited evidence, particularly in Kuwait, has investigated this link during childhood. To address this gap, this study examines the relationship between UPF consumption and ADHD symptom severity in a general sample of children aged 6–12 in Kuwait.

## 2 Materials and methods

A cross-sectional study was designed to examine the relationship between UPF consumption and ADHD symptoms in children. ADHD symptoms were measured using the SNAP-IV Parent Rating Scale, which has been validated in both clinical and community samples (32). UPF consumption was measured using an adapted version of the NOVA-based ultra-processed food questionnaire, which acceptable reliability has been reported in previous studies (33). The questionnaire was available in Arabic only and was distributed online to parents and legal guardians of children between the ages 6 and 12 in Kuwait. Participants were mainly recruited between October and December of 2025 through local community and school online group chats. A brief statement with the objective of the study was included before the questionnaire; all participants consented, and no personal identifying information was collected. Participants were eligible to participate in the study if they were parents or legal caretakers of children aged 6–12, living in Kuwait, and proficient in Arabic. Demographic questions included child age, gender, grade, school type (public or private).

The 26-item SNAP-IV Parent Rating Scale groups behaviors under three behavioral domains: inattention (9 items), hyperactivity/impulsivity (9 items), and oppositional defiant behavior (8 items) (32). For each item, participants were asked to score symptom severity on 4-point scale in the past 6 months. Responses were scored as 0 = Not at all, 1 = Just a little, 2 = Quite a bit, and 3 = Very much. Average rating-per-item for the three subscales were calculated for ease of interpretability resulting in scores ranging from 0 to 3. The NOVA-based ultra-processed food questionnaire was adapted into a 22-item food frequency questionnaire with culturally relevant substitutes where appropriate such as, “ham” and other food items not available in the region (Table 1). “Ready-made salad sauce” was removed as it is not commonly consumed in this target population. Participants were asked to rate the frequency of consumption on a 4-point scale. Each response was scored as follows 0 = Never/Very Rare, 1 = 1–2 times a week, 2 = 3–4 times a week, and 3 = 5 or more times a week.

**Table 1:** Food items of the adapted NOVA-based ultra-processed food frequency questionnaire (22-items).

Item No.	Food item	Response scale
1	Margarine	0 = Never/Very Rare
2	Burger or hotdog bread	1 = 1–2 times a week
3	Soda or energy drinks	2 = 3–4 times a week
4	Biscuits or cookies	3 = 5 or more times a week
5	Packaged snacks or chips	
6	Chocolate bars or candy	
7	Mortadella or deli meat	
8	Sausages, burgers, or nuggets	
9	Flavored yogurt (fruit/chocolate)	
10	Canned or bottled fruit juice	
11	Powdered drink mix (Tang-type)	
12	Ketchup, mayonnaise, or mustard	
13	Ice cream or popsicles	
14	Chocolate milk drink	
15	French fries	
16	Instant noodles	
17	Tea-based bottled drink (iced tea)	
18	Pizza (frozen or restaurant)	
19	Frozen lasagna or ready-made meals	
20	Packaged cake	
21	Cereal bar	
22	Breakfast Cereal	

22-items were adapted from the NOVA-based ultra-processed food questionnaire with minor culturally relevant substitutions (e.g., removal of ham, and ready-made salad sauce). (33) Participants rated consumption frequency as follows: 0 = Never/Very Rare, 1 = 1–2 times a week, 2 = 3–4 times a week, and 3 = 5 or more times a week.

### 2.1 Statistical analysis

Data was collected through Google Forms. Prior to statistical analysis, responses were screened to confirm all eligibility criteria is met, only Kuwaiti parents or legal caretakers of children aged 6–12, residing in Kuwait were included in the sample. After screening, *n* = 46 participants were excluded for not meeting criteria, and a total of *N* = 159 were included in the final sample. IBM SPSS Statistics version 29.0.0.0 was used to conduct Spearman’s correlation to examine the relationship between ADHD symptoms and UPF intake. Internal consistency was assessed using Cronbach’s *α*. Both the adapted NOVA-based UPF questionnaire (Cronbach’s *α* = 0.915) and SNAP-IV subscales (total scale *α* = 0.949, inattention *α* = 0.934, hyperactivity/impulsivity *α* = 0.931, oppositional defiant behavior *α* = 0.945) had high internal consistency. *Z*-scores were used to determine if the dataset has univariate outliers. No data point was over the z-score threshold of ± 3.29. The z-score range for SNAP-IV total scores was (−1.67 to 2.33) and total NOVA-UPF intake was (−2.02 to 3.29), indicating one UPF total score at the borderline, however no extreme outliers were present in the final sample. Correlation coefficients and *p*-values were reported, and statistical analysis was set at *p* < 0.05.

## 3 Results

### 3.1 Demographic characteristics

The survey collected 205 responses in total. 46 participants were removed for not meeting the eligibility criteria. A total of 159 were included in the final study sample. Table 2 summarizes the demographic characteristics of the sample; the mean age of children was 8.58 years (SD = 1.95). Most children were male (59.1%) and attended public schools (76.1%). School grade levels varied with most participants in lower elementary school, specifically 22% in first grade, 13.8% in second grade, and 15.1% in the third grade, respectively.

**Table 2:** Demographic characteristics of sample (*N* = 159).

Characteristic	Mean ± SD
Age	8.58 ± 2.0
Gender	*N* (%)
Female	65 (40.9)
Male	94 (59.1)
School type	
Public	121 (76.1)
Private	38 (23.9)
School Year	
1	35 (22.0)
2	22 (13.8)
3	24 (15.1)
4	19 (11.9)
5	19 (11.9)
6	20 (12.6)
7	18 (11.3)
8	2 (1.3)

### 3.2 SNAP-IV subscales and UPF consumption scores

Table 3 shows the average rating-per-item (ARI) scores for the SNAP-IV subscales and total UPF consumption score. ARI score was highest for hyperactivity/impulsivity 1.29 ± 0.83, followed closely by inattention 1.28 ± 0.72, while oppositional defiant behavior had the lowest score 1.08 ± 0.89. The mean total UPF score was 25.09 ± 12.40 out of a total possible score of 66.

**Table 3:** Average rating-per-item scores for SNAP-IV subscales and UPF intake score.

Variable	Mean ± SD
Inattention	1.28 ± 0.72
Hyperactivity/impulsivity	1.29 ± 0.83
Oppositional defiant behavior	1.08 ± 0.89
Total UPF consumption score	25.09 ± 12.40

SNAP-IV average rating per item score range 0–3. Total UPF score range = 0–66.

### 3.3 Correlation analysis of ADHD symptoms and UPF consumption

**Figure 1:**
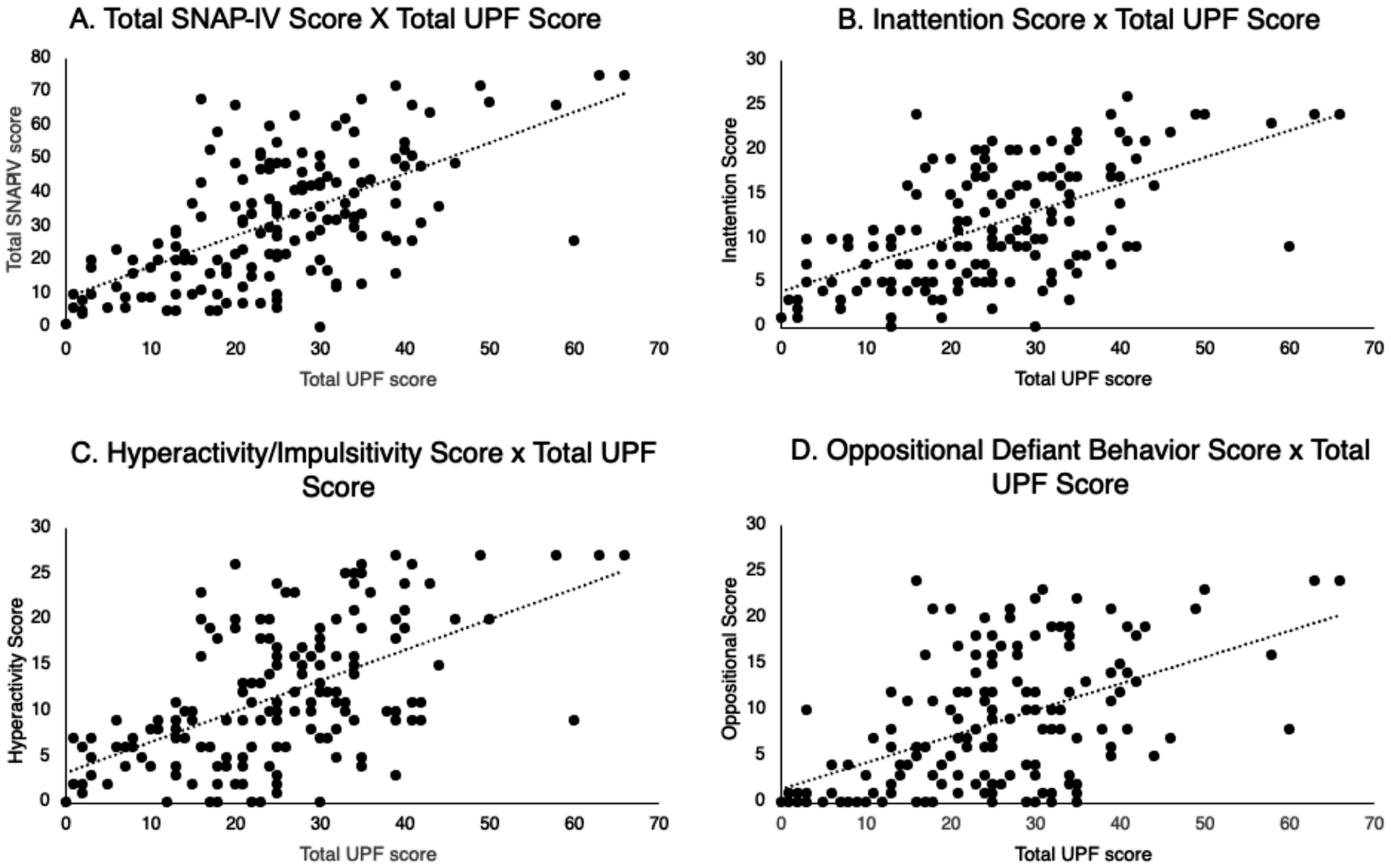
Scatter plots displaying spearman correlations between total UPF intake score and SNAP-IV subscale domains: **(A)** total SNAP-IV score, **(B)** inattention, **(C)** hyperactivity/impulsivity, and **(D)** oppositional defiant behavior.

Spearman’s correlation analysis revealed a statistically significant positive correlation between UPF intake and ADHD symptom severity (Table 4). After adjusting for age, the correlation between NOVA-UPF score and total SNAP-IV score remained positive [*ρ* = 0.576, 95% CI (0.463, 0.673) *p* < 0.001] (Table 4, Figure 1A). All correlations represented large effect sizes (Fisher’s *z* = 0.659). Significant positive correlations were observed across all SNAP-IV behavioral domains, in order of strongest to least: hyperactivity/impulsivity (*ρ* = 0.564, *p* < 0.001), followed by inattention (*ρ* = 0.546 *p* < 0.001), and oppositional defiant behavior, which showed a relatively lower though still significant correlation (*ρ* = 0.482, *p* < 0.001) (Table 4, Figures 1A–D). For boys (*n* = 94) consistently stronger positive associations were observed across all behavioral domains compared with girls (*n* = 65) (Table 5). The age-adjusted correlation with total symptoms was notably high in boys (*ρ* = 0.665, *p* < 0.001) compared to a significantly positive though relatively moderate association among girls (*ρ* = 0.439, *p* < 0.001). Further analysis by SNAP-IV domains revealed that for boys, hyperactivity/impulsivity (*ρ* = 0.657, *p* < 0.001) had the strongest relationship with UPF intake, while, among girls, inattention, (*ρ* = 0.457, *p* < 0.001) had the highest correlation across SNAP-IV behavioral domains. Collectively, our findings demonstrate a positive relationship between UPF consumption and behavioral symptoms of ADHD after adjusting for age.

**Table 4:** Correlations between UPF consumption and SNAP-IV scales (*N* = 159).

Variable	*ρ*	*p*-value^a^	95% CI
Total SNAP-IV score	0.576	<0.001	[0.463, 0.673]
Inattention	0.546	<0.001	[0.427, 0.647]
Hyperactivity/impulsivity	0.564	<0.001	[0.442, 0.661]
Oppositional defiant behavior	0.482	<0.001	[0.353, 0.593]

^a^Spearman’s partial correlations conditioned on age significance *p* < 0.05. CI, confidence interval.

**Table 5:** Correlations between UPF consumption and SNAP-IV scales by gender.

Variable	Boys (*n* = 94) *ρ* (*p*-value*^a^*)	Girls (*n* = 65) *ρ* (*p*-value*^a^*)
Total SNAP-IV score	0.665 (<0.001)	0.439 (<0.001)
Hyperactivity/impulsivity	0.657 (<0.001)	0.372 (0.002)
Inattention	0.618 (<0.001)	0.457 (<0.001)
Oppositional defiant behavior	0.552 (<0.001)	0.332 (0.007)

Boys (*n* = 94) and girls (*n* = 65). Spearman’s partial correlations conditioned on age significance *p* < 0.05.

## 4 Discussion

### 4.1 Main findings

The aim of this study was to examine the relationship between UPF consumption and ADHD behavior among a general sample of children in Kuwait. Our results show a clear, significantly positive association between UPF consumption and ADHD behavioral symptoms. Further analysis revealed that, in the total sample, hyperactivity/impulsivity had the strongest correlation with UPF intake. The relationship between UPFs and hyperactivity was also more pronounced in boys compared to girls. One possible behavioral explanation is that this domain is most noticeable compared to the other behavioral domains making it easier for parents to identify and report.

However, several proposed mechanisms may explain why hyperactivity/impulsivity was more strongly associated with UPF intake in this sample compared to other ADHD domains. Refined sugars used in UPFs may impair cognitive function and inhibitory control through glycemic dysregulation and insulin resistance (34). Beyond refined sugars, synthetic food dyes have recently gained attention for their potential to interfere with neurochemical signalling disrupting pediatric neurological development (35, 36). The gut microbiome brain axis is increasingly being recognized as a potential driver of neurodevelopmental disorders (37). Synthetic additives and emulsifiers found in UPFs have been suggested to disrupt the gut microbiome by increasing intestinal permeability and releasing pro-inflammatory molecules in the blood–brain barrier, which could impact behaviors such as hyperactivity and impulse control (38). However, the nature of our study cannot provide direct evidence for the proposed biological pathways. Rigorous dietary interventional studies where UPF consumption is reduced or eliminated systematically over time is needed to determine if ADHD symptoms persist or change with UPF consumption. Prospective cohort studies starting from early childhood are also needed in the Kuwaiti pediatric population to determine whether ADHD symptoms present before increased UPF consumption or vice-versa, this would help establish the temporal direction of this relationship. Lastly, studies examining the gut-brain axis directly through gut microbiome sequencing could elucidate how synthetic ingredients found in UPFs disrupt the gut microbiome and contribute to neuroinflammation over time.

Interestingly while boys in our sample showed stronger associations with hyperactivity/impulsivity, the strongest correlation among girls was observed with inattention (Table 5). This observation opens a broader discussion on ADHD gender-related differences. ADHD is more prevalent among boys during childhood; however, this may partially be the result of boys being more likely to get diagnosed for presenting more “externalizing” behaviors such as hyperactivity and impulsivity (39, 40). In contrast girls tend to present “internalizing” behaviors such as inattention that may go undetected. Although these results are from a general population sample, these findings reflect broader gender related differences observed with ADHD symptom presentation. More research is needed to determine whether the relationship between UPF intake and ADHD behavioral symptoms differs by gender.

### 4.2 Comparisons

The strong positive association between UPF intake and ADHD symptom severity aligns with a growing body of international research. For instance, a recent study demonstrated that children aged 6–11 with an ADHD diagnosis (*n* = 111) consumed significantly higher UPFs compared to their peers (*n* = 1,024) that were not diagnosed (10). This association remained statistically significant even after controlling for other cofounding variables. Similarly, longitudinal results from the Canadian CHILD cohort study found that higher UPF consumption at age 3 significantly predicted both hyperactivity and inattention by age 5, however these results did not significantly differ by gender (41). Further longitudinal evidence from a Brazilian cohort suggests that higher UPF intake between the ages 3 and 4 was a significant predictor of hyperactivity and inattention at 12–13 years old, however no differences in gender were observed (42). Together, these findings from both cross-sectional and longitudinal study designs support the association between UPF consumption and ADHD behavioral symptoms in children. However, in contrast to the studies mentioned, our results point to a pattern of association that differs by gender, with boys having stronger correlations, despite no reported gender difference in ADHD prevalence in the Arab Gulf region (4). Our results also contribute to the limited data available on ADHD and UPFs in the region, however much more regional data is needed to confirm these results.

### 4.3 Strengths and limitations

This is the first study, to our knowledge, to examine the relationship between UPF intake and ADHD behaviors in children in Kuwait. One major strength of our study is the use of the SNAP-IV Parent Rating Scale to measure ADHD-related behavior. However, some limitations need to be considered. Namely, the reliance on parent-reported data introduces recall bias that limits the generalizability of conclusions to other populations. Additionally, potential confounders such as, nutritional status, physical activity, and socioeconomic factors were not collected and therefore could not be adjusted for in this analysis. Future longitudinal studies, larger samples, and more detailed dietary assessment tools are needed to confirm these results.

Another major drawback of these results is the inability to establish causal direction. ADHD-related behaviors such as, impulsivity can also contribute to UPF intake indirectly through disordered eating patterns (43). As much as 20% of children with ADHD are estimated to develop eating disorders, with binge eating disorder being the most common (43). One possible explanation is the use of UPFs as a coping mechanism or a reward to stimulate dopamine signalling typically altered in ADHD (9). Together, these mechanisms suggest a feedback loop between ADHD behaviors and UPF consumption. UPF intake may exacerbate ADHD symptom severity, which may then increase preference for UPFs.

### 4.4 Conclusion

In essence, our results reinforce the positive relationship between UPF consumption and ADHD symptoms in children between the ages 6 and 12 years in Kuwait. Our findings along with previous international studies demonstrate how ultra-processed diets are strongly linked to ADHD behaviors during development. Beyond neurodevelopment, UPFs have been consistently proven to harm metabolic health, contributing to insulin sensitivity and the progression of non-communicable diseases. Therefore, although these results do not prove causation, the strength of the association observed (*ρ* = 0.576, *p* < 0.001) highlights the urgent need for public policy change regulating UPFs in Kuwait, especially items marketed towards children, and reassess the safety of synthetic food dyes and other artificial ingredients found in UPFs. Targeted nutritional interventions are needed to address the high UPF consumption observed in Kuwait. Nutritional education aimed at parents and caregivers has demonstrated success, a recent randomized clinical trial showed that nutritional epigenetics education for parents of children with ADHD or autism significantly reduced UPF consumption (44). Future public health efforts in Kuwait should consider integrating nutritional epigenetics into health curriculums for students and parents to benefit.

## Data Availability

The raw data supporting the conclusions of this article will be made available by the authors, without undue reservation.
